# Development of a readout circuit and platform for uric acid measurement for urolithiasis application

**DOI:** 10.3389/fbioe.2026.1748462

**Published:** 2026-06-04

**Authors:** Meo Vincent Caya, Wen-Yaw Chung, Hsing-Chung Liang, Rih-Lung Chung, Yen-Wei Liu, Roozbeh F. Ramezani, Vincent F. S. Tsai

**Affiliations:** 1 Department of Electronics Engineering, Chung Yuan Christian University, Zhongli, Taiwan; 2 School of Electrical, Electronics, and Computer Engineering, Mapua University, Manila, Philippines; 3 Department of Urology, Min-Sheng General Hospital, TaoYuan, Taiwan

**Keywords:** amperometric, electrochemical, kidney stone, multi-parameter sensing, uric acid, urolithiasis

## Abstract

**Introduction:**

Uric acid (UA) is a clinically relevant urinary biomarker for assessing the risk of urolithiasis, but portable electrochemical platforms require wide dynamic range, robust bio-electronic interfacing, and validation with real samples. In this study, we present a Complementary Metal-Oxide-Semiconductor (CMOS) potentiostatic amperometric readout circuit and a portable sensing platform for urinary UA measurement.

**Methods:**

The proposed circuit is designed to work with both two- and three-electrode electrochemical configurations and uses separate amplifier loops for electrode bias control and current readout to improve stability and linearity during measurement. Fabricated in a 0.18-μm CMOS process, the readout integrated circuit occupies an active area of 102 μm × 195 μm and operates from a 3.3 V supply while maintaining an oxidation potential of approximately 0.7 V at the sensing interface. DC simulation was performed to evaluate the current detection capability of the architecture. Fabricated silicon measurements were conducted to validate circuit operation, and the system was evaluated using UA assays and fresh urine samples under controlled dilution. The readout circuit was further implemented within a portable multi-parameter urine sensing platform that supports concurrent measurement of UA, pH- and calcium-related signals, and conductivity with microcontroller-based digitization.

**Results:**

DC simulation indicates that the proposed architecture can theoretically support a current detection range from 150 pA to 160 μA (>5 decades) with less than 2% current replication error under nominal conditions. Fabricated silicon measurements validate stable potentiostatic operation and linear electrochemical response within the experimentally evaluated UA concentration range (20–500 ppm), demonstrating a functional electrochemical interface of the readout circuit. The multi-decade current capability therefore represents a simulation-supported design potential of the architecture, while the experimental validation focuses on the sensing range of the implemented UA assay. When evaluated using fresh urine samples under controlled dilution, the system exhibits a clear linear relationship between readout current and UA concentration over a relevant range of 20–500 ppm, with measurement differences typically within 10 ppm and less than 5% compared with an adjusted commercial analyzer.

**Conclusions:**

Together, these results demonstrate a compact and robust electrochemical readout solution that supports flexible sensor integration and provides a practical foundation for portable, multi-biomarker urine analysis and future data-driven monitoring of urolithiasis risk.

## 1 Introduction

Nephrolithiasis (urolithiasis) refers to the formation of urinary stones within the kidney and urinary tract and is strongly associated with metabolic conditions such as diabetes mellitus, obesity, hyperlipidemia, and gout (Thakore P. and Liang TH, 2023). These conditions influence urinary composition and promote lithogenic environments, including increased uric acid (UA) and calcium excretion. Epidemiological studies report that urolithiasis affects approximately 1%–19.1% of the population across Asia, with recurrence rates of 21%–53% within 3–5 years, highlighting the importance of the continuous monitoring of urinary biomarkers (Liu et al., 2018). In Taiwan, population-based studies report a lifetime prevalence of 9.6% and recurrence rates increasing to 34.71% within 5 years, further highlighting the need for the continuous monitoring of urinary biomarkers (Lee et al., 2002; Yu et al., 2016).

Among the various urinary stone types, UA stones account for approximately 5%–20% of all cases and are closely associated with hyperuricosuria, low urinary pH, and reduced urinary volume (Hall, 2009; Liu et al., 2018; Ma et al., 2018). The prevalence of UA stones has increased in recent years due to dietary and environmental factors, as well as metabolic disorders such as obesity and diabetes (Ma et al., 2018). These trends emphasize the need for practical methods for monitoring UA levels in urine for the improved assessment and management of urolithiasis risk.

Monitoring urine composition, including UA concentration, pH, calcium, and other related parameters, plays a vital role in the analysis and prevention of urolithiasis. Several approaches have been explored for urine analysis, including multi-parameter sensing (Chung et al., 2020; Shimohigoshi and Karube, 1996; Yaroshenko et al., 2015) and single-parameter detection methods (Porowski et al., 2013; Silverio et al., 2016). In particular, electrochemical sensing platforms have demonstrated strong potential for the rapid and cost-effective analysis of urinary ionic composition, with good agreement compared to reference laboratory methods when combined with appropriate signal processing technique (Yaroshenko et al., 2015).

Although several electrochemical sensing platforms and commercial analog front ends (AFEs) (Texas Instruments, 2014) have been reported, most existing designs are general-purpose and do not explicitly target the circuit-level requirements of UA amperometric sensing. In particular, UA sensing in urine presents specific circuit-level challenges, including wide variation in sensing current due to concentration-dependent enzymatic reactions, time-varying electrode impedance in biologically complex matrices, and the need for stable WE–RE bias control under non-ideal electrochemical loading conditions. Existing general-purpose AFEs are typically designed for broad applicability and often rely on discretized bias settings and tightly coupled potentiostatic control and readout paths, which can limit continuous dynamic range and reduce measurement robustness under varying sensor impedance. As a result, these architectures may not fully address the requirements for stable and accurate current measurement in urine-based UA sensing applications. Therefore, there is a need for a readout architecture that provides continuous analog bias control, improved tolerance to impedance variation, and reliable current-domain signal acquisition for biologically relevant sensing environments.

Although current-mirror-based potentiostats and regulated bias architectures have been extensively studied—including current-mirror topology (Ahmadi and Jullien, 2009), array-oriented regulated bias designs (Ayers et al., 2007), and wide-dynamic-range Complementary Metal-Oxide-Semiconductor (CMOS)implementations (Wang et al., 2010) —such research primarily emphasizes array scalability, low-noise detection, or general-purpose amperometric sensing and does not explicitly address the circuit-level challenges associated with enzymatic UA sensing in biologically complex matrices such as urine. In addition, our prior multi-parameter readout integrated circuit (IC) (Silverio et al., 2020) focused on multi-modal integration and cross-talk mitigation rather than stable potentiostatic operation and wide-range current sensing under impedance-varying biological conditions.

In contrast, this study provides an application-driven refinement of CMOS potentiostatic readout architecture, targeting the specific requirement of maintaining stable WE–RE bias under time-varying electrode impedance while supporting multi-decade current variation encountered in enzymatic UA sensing. This is achieved through (i) explicit architectural partitioning of bias regulation and current readout using a dual-operational transconductance amplifier (OTA) feedback structure and (ii) incorporation of an Rf–Cf compensation network to enhance loop stability against electrode impedance variation and transient electrochemical effects.

Unlike prior general-purpose designs, the proposed architecture is specifically configured to improve electrochemical interface robustness in urine-based sensing environments, where electrode impedance and reaction dynamics vary over time. The resulting architecture supports a simulated current span of approximately 150 pA–160 µA and is validated through silicon implementation and urine-based UA measurements used as a biologically relevant test case for evaluating circuit-level electrochemical interfacing performance. The primary contribution of this study is, therefore, not the introduction of a fundamentally new potentiostat topology but the application-specific architectural adaptation and system-level validation of a CMOS readout circuit for stable amperometric sensing in biologically complex environments. This distinction clarifies the contribution of this study as a system-oriented advance in electrochemical readout design for biologically relevant sensing applications.

This study focused on the development and application of a current-type readout circuit for sensing UA in urine. After the chemical reaction producing current is converted into UA concentration through a linear relationship, it is then compared with commercial instruments for UA detection to evaluate the performance of the proposed UA detection system. The measurement range for a UA detection system was between 20 ppm and 500 ppm, with ppm-level concentration monitoring based on the calibration slope between current and concentration. The UA detection system was implemented using an amperometric readout circuit that was designed using IC utilizing 0.18 um technology and incorporated in a microcontroller development platform device to determine the value of UA.

This study makes several key contributions. A CMOS potentiostatic amperometric readout architecture based on a dual OTA topology is presented, in which the working-reference electrode (RE) bias control is explicitly separated from the current readout path to improve electrochemical stability and robustness against sensor impedance variations. The proposed current-mode readout circuit is implemented in a 0.18-µm CMOS process and supports a simulated current range from 150 pA to 160 µA (>5 decades) with a relative error below 2% under direct current (DC) analysis while maintaining a stable oxidation potential at the sensor interface. Experimental validation using real urine samples shows a linear response over a relevant UA concentration range of 20–500 ppm with performance comparable to that of a certified commercial analyzer. In addition, the scalability of the proposed architecture is demonstrated through integration into a portable multi-parameter urine-sensing platform, highlighting its applicability to precision biosensing in biologically complex environments. In this context, the urine-based UA sensing experiments are primarily intended to demonstrate the practical electrochemical interfacing capability of the proposed CMOS readout circuit under enzymatic amperometric sensing conditions rather than to establish analytical selectivity or optimize the electrochemical assay itself.

## 2 Materials and methods

### 2.1 Electrochemical sensing principle and experimental methodology

In this study, a manufacturer-calibrated commercial uric acid (UA) test strip (ET-301, Bioptik Technology, Inc.) was used for electrochemical validation. The commercial strip operates on an enzymatic hydrogen peroxide generation mechanism, where UA is oxidized by uricase and the resulting H_2_O_2_ is detected amperometrically at an applied WE–CE potential of approximately 0.7 V. The strip incorporates defined working and RE channels and a capillary-based fixed-volume (∼1 μL) sample loading structure to improve repeatability. The ET-301 device used in this study was adjusted by the manufacturer for urine-based measurement, with a specified detection range of 2–50 mg/dL. The commercial strip was interfaced with the proposed potentiostatic readout circuit to evaluate the electrical performance and bias stability of the CMOS readout architecture under enzymatic amperometric operation in a urine matrix. Accordingly, the electrochemical assay chemistry and operating potential follow the manufacturer-calibrated characteristics of the commercial strip, while the focus of this study is the electrical and circuit-level validation of the proposed CMOS potentiostatic readout architecture.

UA is detected in urine by its oxidization with UA enzyme in aqueous solution (Equations 1 and 2). H_2_O_2_ releases electrons after the oxidation reaction. In the three-electrode system (Chung et al., 2018), it can detect current changes in the system.

C5H4N4O3+2H2O+O2 → C2H6N4O3+H2O2+CO2, (1)

H2O2 → O2+2H++2e‐. (2)

### 2.2 Readout circuit and system implementation

**FIGURE 1:**
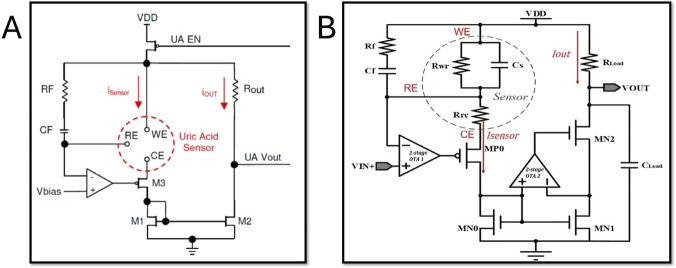
**(A)** Simplified readout circuit for uric acid sensing. **(B)** Proposed readout circuit for uric acid sensing.

As shown in Figure 1, this study employs a CMOS potentiostatic readout circuit for amperometric sensing (Silverio et al., 2020; Chung et al., 2011). The electrochemical sensor uses a standard three-electrode configuration consisting of the RE, working electrode (WE), and counter electrode (CE). A potentiostat maintains a fixed potential between the WE and RE, biasing the electrochemical cell at the oxidation potential obtained from cyclic voltammetry (CV). Under this condition, a redox current flows from the WE to the CE, and its magnitude is proportional to the UA concentration.

Figure 1B shows the complete potentiostatic readout architecture proposed here. In this design, the electrochemical cell is modeled using resistive and capacitive elements to represent electrode and interface effects. Two dedicated OTAs are used to separate the WE–RE biasing loop from the current readout path, which improves loop stability, reduces sensitivity to sensor impedance variations, and improves linearity. Figure 1A shows a simplified implementation of this architecture, where the potentiostatic control and current readout are combined into a single loop. In this architecture, the electrochemical signal is represented by the current generated at the WE as a result of UA oxidation. The potentiostat loop, formed by the operational amplifier, transistor M3, and the RE and CE, enforces a constant WE–RE potential, which is a fundamental requirement of amperometric sensing. By fixing this electrode potential, the circuit ensures that the measured signal is the reaction current, the magnitude of which is proportional to the UA concentration.

The readout is implemented in the current domain wherein the amperometric sensor current is sensed at the WE, mirrored through transistors M1 and M2, and subsequently converted into a voltage across the load resistor Rout, producing the output voltage (UA V_out_). Because the electrode voltage is regulated while the output depends on the measured current, the circuit clearly operates as an amperometric readout. This simplified circuit provides a compact and low-complexity solution and was used for the initial implementation and validation of UA sensing with actual urine samples. After the oxidation potential is applied, the sensing current is mirrored to the output stage and converted into a voltage across a load resistor, given by (Equation 3):

VOUT=VDD‐Isensor * Rload, (3)

where I_sensor_ is directly proportional to the UA concentration.

### 2.3 Measurement setup and urine sample preparation

**FIGURE 2:**
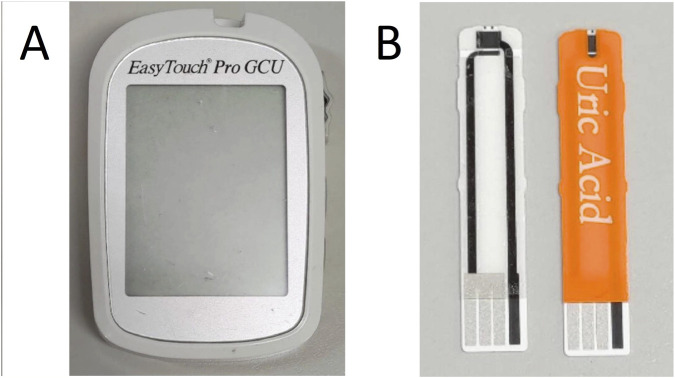
**(A)** Commercial uric acid measurement device ET-301. **(B)** Commercial uric acid test strip.

The output voltage obtained by the UA readout circuit is converted into concentration (ppm) and compared with a commercially available multifunctional detection system for blood glucose/UA/cholesterol provided by Bioptik Technology, Inc. product model ET-301, a medical device in Taiwan, registration number 003204. Figure 2A shows the commercially available ET301 device that was used for comparison with the proposed UA concentration measurement device for urine. Figure 2B shows the test strips that were used for both the ET301 and the proposed UA detection device.

In the initial implementation and experimental validation of the UA readout system, the simplified circuit (Figure 1A) was used for testing. Firstly, an ET-301 analyzer and a commercial UA test strip (Figures 2A,B, respectively) were employed to measure the UA concentration in urine as a reference. The simplified UA readout circuit was then interfaced with an NI USB-6210 data acquisition instrument to continuously monitor the output voltage Vout during urine testing.

**FIGURE 3:**
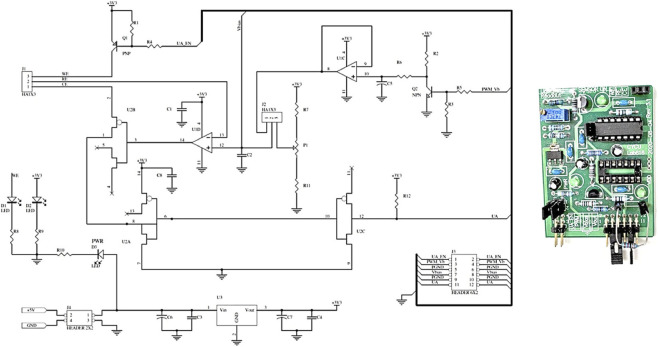
Schematic-level representation of the PCB-based uric acid readout system based on the simplified readout circuit in Figure 1A used for initial experimental validation.

Figure 3 illustrates the schematic-level implementation of the printed circuit board (PCB)-based UA readout system used for initial experimental validation. This board-level circuit implements the simplified potentiostatic amperometric architecture shown in Figure 1A, in which the WE–RE bias control and current readout functions are combined into a single feedback loop. The schematic highlights the functional signal paths, including the electrode interfaces, biasing network, current readout stage, and external measurement connections, to the data acquisition system. This PCB-based realization was used to validate the sensing principle and urine measurement protocol prior to full integration of the refined dual-loop architecture (Figure 1B).

**FIGURE 4:**
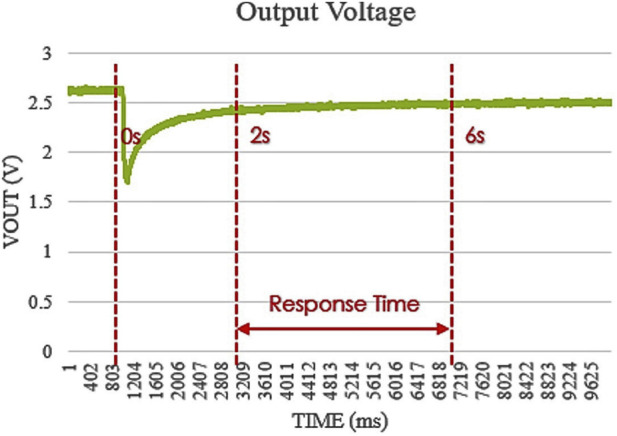
Output voltage of the uric acid reading circuit.

Upon urine titration, an oxidation reaction occurs between the analyte and the enzyme in the test strip, generating a sensing current that produces a corresponding voltage drop at V_out_. This abrupt voltage transition marks the onset of the electrochemical reaction. Accordingly, the system monitors V_out_ within a 2–6 s timeframe following this event to extract the steady-state voltage used for UA quantification (Figure 4). Urine samples were freshly collected from two independent volunteer donors under institutional ethics approval and used as a biological matrix for circuit-level validation. Samples were analyzed separately and were not pooled. Controlled UA concentration levels spanning the evaluated sensing range (20–500 ppm) were generated by diluting the collected samples, producing approximately ten concentration points used for calibration and comparison with the commercial ET-301 analyzer. The objective of these experiments was to evaluate readout circuit performance under urine-based sensing conditions rather than to establish clinical diagnostic metrics.

## 3 Results and discussion

### 3.1 Relationship equation between uric acid concentration and current

**FIGURE 5:**
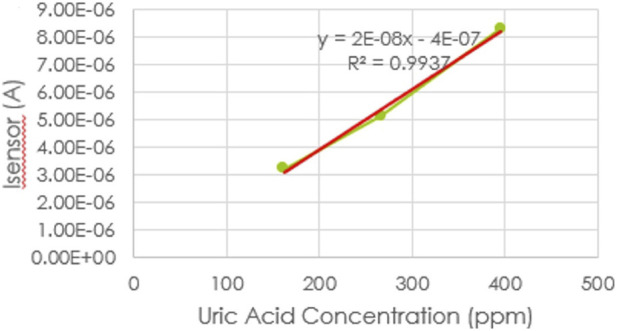
Comparison chart of average current at different concentrations.

Table 1 presents three repeated measurements at different UA concentrations. The corresponding coefficient of determination (R^2^) values are 0.9837, 0.9987, and 0.9851 for the first, second, and third readouts, respectively, compared with the ET-301 reference measurements. All R^2^ values exceeded 0.98, indicating strong linear agreement between the proposed readout circuit and the reference device. In addition, the averaged readout current yielded an R^2^ greater than 0.99 (Figure 5), further confirming the high correlation between the measured current and UA concentration.

**TABLE 1:** ET301 measurement and UA readout output current.

ET301 (ppm)	First UA readout (µA)	Second UA readout (µA)	Third UA readout (µA)	Ave UA readout (µA)
162	3.25	3.26	3.11	3.21
267	5.14	5.36	4.86	5.12
396	8.88	7.64	8.25	8.26

The relationship between UA concentration and measured current was evaluated over a concentration range of 20–500 ppm. To establish the calibration model, multiple measurements were performed across this range using the proposed readout circuit. Table 2 shows the urine test measurements obtained from diluted urine samples derived from two independent donors, generating ten concentration points spanning the evaluated sensing range (20–500 ppm).

**TABLE 2:** Multiple concentration measurement between 20–500 PPM.

ET-301 (ppm)	UA readout (V)	UA current (µA)
70.2	3.2335	2.015
182.6	3.1441	4.724
259.0	3.1053	5.900
267.5	3.1023	5.991
310.3	3.0625	7.197
396.3	3.0274	8.261
411.3	3.0060	8.909
437.0	2.9800	9.697
455.6	2.9744	9.867
484.3	2.9461	10.724

**FIGURE 6:**
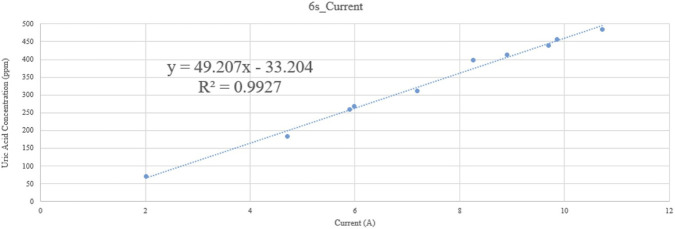
Uric acid concentration–current relationship equation.

Figure 6 shows the plotted diagram based on Table 2, having multiple measurements between 20–500 ppm using the ET-301 commercial device and the proposed UA readout circuit. The diagram yields a linear equation (Equation 4):

$$y\left(\text{UA Concentration}\right)=49.207\;*\;x\;\left(\text{UA current}\right)\;‐\;33.204.$$ (4)

The regression parameters were obtained using ordinary least squares (OLS) linear regression without weighting, using the UA concentration measured by the ET-301 analyzer as the reference variable.

Using the derived linear relationship, the measured current was converted into UA concentration and compared with the ET-301 reference values to evaluate accuracy. As summarized in Table 3, the maximum absolute error is 23 ppm, corresponding to a maximum relative error of 9.12%. These values are within the typical 15% error margin specified for commercial instruments, indicating acceptable agreement within the tested range.

**TABLE 3:** Uric acid concentration (UA Readout) comparison table with commercial instruments.

ET-301 (ppm)	UA readout (ppm)	ET301-UA readout (ppm)	ABS (ET301-UA Readout)/ET301
70.2	66.0	4.2	6.05%
182.6	199.3	−16.7	9.12%
259.0	257.1	1.9	0.73%
267.5	261.6	5.9	2.21%
310.3	320.9	−10.6	3.43%
396.3	373.3	23.0	5.81%
411.3	405.2	6.1	1.49%
437.0	444.0	−7.0	1.59%
455.6	452.3	3.3	0.72%
484.3	494.5	−10.2	2.11%

### 3.2 Integrated circuit simulation results

For the on-chip implementation, the proposed UA readout architecture shown in Figure 1B was designed using the UMC 0.18 µm CMOS process. DC analysis was conducted in HSPICE by sweeping the sensor interface resistance R_wr_ to evaluate the operating current range under varying electrochemical conditions.

**FIGURE 7:**
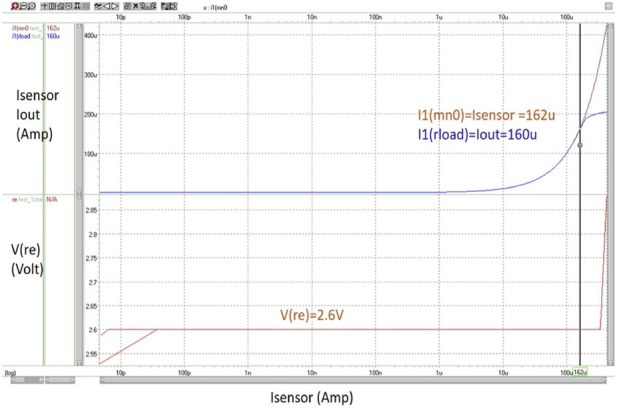
DC simulation results showing output current accuracy and reference electrode voltage regulation as the sensing current I_sensor_ is swept in the microampere range.

Figure 7 presents the circuit response in the high-current operating region. The injected sensing current I_sensor_ is swept up to the microampere range, and the resulting output current I_out_ is compared with the input while monitoring the (RE) voltage V_re_. The simulation results show that V_re_ remains tightly regulated at 2.6 V over the entire sweep, confirming stable potentiostatic operation. At an input current of I_sensor_ = 162 µA, the simulated output current is I_out_ = 160 µA, corresponding to a relative error below 2%.

**FIGURE 8:**
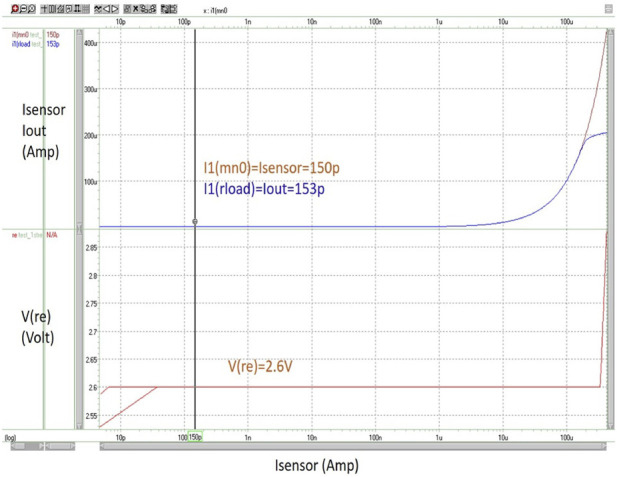
DC simulation results showing output current accuracy and reference electrode voltage regulation as the sensing current I_sensor_ is swept in the picoampere range.

Figure 8 shows the circuit behavior in the low-current regime, where I_sensor_ is swept down to the picoampere level. Despite the extremely small signal magnitude, the RE voltage remains well regulated, and the readout path preserves good linearity. For an input current of I_sensor_ = 150 pA, the output current is I_out_ = 153 pA, resulting in a relative error of less than 2% under DC simulation. This indicates that the proposed architecture can potentially operate across a current range from 150 pA to 160 µA (>5 decades) under DC simulation conditions, while maintaining stable RE biasing.

The wide current range of 150 pA to 160 µA is supported by DC simulation of the proposed architecture, which evaluates current replication accuracy and RE bias stability across several decades of input current. The fabricated silicon measurements presented in this study primarily validate stable potentiostatic biasing and linear electrochemical response within the experimentally evaluated UA concentration range corresponding to the implemented urine sensing assay. A full multi-decade experimental current sweep, minimum resolvable current characterization, and detailed noise floor analysis were not the primary focus of the present study. Therefore, the >5-decade current span should be interpreted as a simulation-demonstrated capability of the circuit architecture rather than a fully experimentally characterized system-level measurement range.

### 3.3 Circuit operation and bias stability analysis

In contrast to the simplified single-loop design in Figure 1A, which focuses on compact implementation, the Figure 1B architecture explicitly models the electrochemical interface using R_wr_, R_rc_, and Cs and separates potentiostatic control from current readout using dual OTAs. This approach is designed to improve WE–RE bias accuracy and enhance robustness to sensor impedance variations and transient effects through architectural separation of bias control and current readout while supporting a wide dynamic range exceeding five orders of magnitude under simulation conditions. These are achieved through the combined use of closed-loop potentiostatic control and current-mode readout. A high-gain feedback loop formed by OTA1, MP0, and the RE–CE path continuously adjusts the CE voltage to maintain the RE potential at the desired bias level, thereby stabilizing the WE–RE voltage despite changes in solution resistance and electrode interface properties. The inclusion of an Rf–Cf feedback network improves tolerance to sensor impedance variations and transient phenomena by limiting the loop bandwidth and attenuating rapid disturbances caused by double-layer capacitance and abrupt electrochemical changes. In addition, the use of current mirroring for signal acquisition enables wide dynamic-range sensing by isolating the WE from the output stage and allowing flexible current-to-voltage conversion through the load resistor, ensuring accurate measurement across varying analyte concentrations without compromising electrochemical stability.

Although the refined architecture increases circuit complexity, it is designed to improve electrochemical fidelity and current acquisition reliability, which are critical for accurate amperometric biosensing. These improvements are achieved through precise control of the sensor bias and current-domain signal processing. A high-gain feedback loop formed by OTA1, MP0, and the RE–CE path continuously regulates the RE potential, maintaining a stable WE–RE voltage despite variations in electrode impedance and solution conditions. The resulting sensing current is then mirrored using an OTA-assisted current mirror, enabling accurate signal replication while electrically isolating the electrochemical interface from the output circuitry.

### 3.4 Silicon measurement results

**FIGURE 9:**
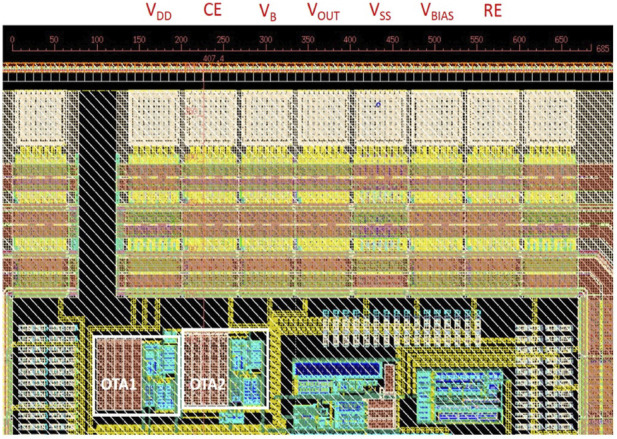
Uric acid readout circuit chip photomicrograph (500 × 407.4 μm).

**FIGURE 10:**
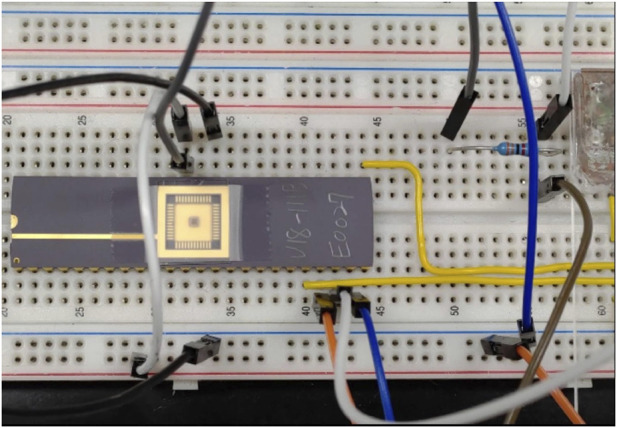
Integrated circuit under test for uric acid concentration measurement.

Figure 9 shows the photomicrograph of the fabricated UA readout IC, including I/O pads; the active readout core occupies 102 µm × 195 µm (pads excluded). The chip is powered from VDD = 3.3 V; a 2.6 VBIAS is applied to the non-inverting input VIN+ (VBIAS) of the two-stage OTA to establish the potentiostatic operating point. Under these bias conditions, a WE–CE potential of approximately 0.7 V is produced, setting the intended electrochemical oxidation point for current-domain sensing; the resulting sensing current I_sensor_ is routed to the output measurement node. The external wiring and measurement configuration used during silicon characterization are depicted in Figure 10.

Table 4 shows the UA Readout Integrated Circuit test measurement using three UA concentrations with the corresponding PPM measurement based on the linearity equation formula using the current output from the UA Readout IC. It also shows that the difference between the ET301 and the measured PPM was within 10 PPM with a percent error of less than 5%.

**TABLE 4:** Comparison table between UA Readout IC measurement results and commercial device (ET301).

ET-301 (PPM)	I (µA)	UA IC readout (ppm)	Difference	% Error
373.2	8.14	367.34	5.86	1.57%
322	7.39	330.44	−8.44	−2.62%
240.8	5.39	232.02	8.78	3.65%

### 3.5 Discussion and comparison with commercial AFEs

Commercial electrochemical AFEs offer compact and low-power solutions for portable and point-of-care sensing systems; they are widely used due to their ease of integration and digital configurability. Devices such as the LMP91000 are designed as general-purpose interfaces and are well suited to rapid prototyping and chemical or gas sensing applications. To support a broad range of sensors, these AFEs typically employ discretized WE–RE bias levels and closely coupled potentiostatic control and readout paths. Although this approach simplifies system implementation, it can limit continuous dynamic range and reduce measurement robustness when applied to biological fluids that exhibit variable electrode impedance and time-varying electrochemical behavior.

The proposed CMOS amperometric readout IC was developed to meet the specific requirements of UA sensing in urine, where stable electrode biasing, accurate low-current measurement, and wide dynamic range are critical for reliable analyte quantification. The design employs a dual OTA architecture that separates the WE–RE bias control loop from the current readout path. This separation enables continuous analog potentiostatic control while minimizing loading of the electrochemical interface by the output circuitry. As a result, the RE potential remains well-regulated despite changes in sensor impedance and transient electrochemical effects commonly encountered in urine-based measurements.

Table 5 compares the key performance metrics of the proposed readout IC with a representative commercial AFE. While commercial solutions such as the LMP91000 provide advantages in terms of ultra-low-power operation and digital programmability, the proposed design focuses on architectural flexibility for electrochemical interfacing and continuous analog bias control. The comparison is intended to highlight design trade-offs rather than to provide a direct experimental benchmark between the two approaches. As shown in Table 5, DC simulation supports less than 2% current replication error across an input current range from 150 pA to 160 µA (> 5 decades), indicating the potential capability of the architecture to support multi-decade current sensing. Silicon measurements confirm stable WE–RE bias regulation and linear sensing behavior within the experimentally tested UA concentration range. Together, these results indicate that the proposed architecture can maintain stable potentiostatic operation in silicon, while the multi-decade current span remains a simulation-supported architectural capability.

**TABLE 5:** Comparison between commercial electrochemical AFE and proposed CMOS readout IC.

Metric	Proposed work	LMP91000
Supply voltage	3.3 V	2.7–5.25 V
Average supply current	Application-driven (not ultra-low-power optimized)	<10 µA (3-lead mode, typ.)
WE–RE bias voltage	2.6 V (continuous, external vbias)	±1%–24% of VREF or VDD (discrete steps)
Bias resolution	Continuous (OTA-limited)	1%–2% programmable steps
Reference electrode bias error	<2% over 150 pA–160 µA (DC simulation)	Not explicitly specified in datasheet
Open-loop gain (bias control)	Two-stage OTA; >100 dB (design target)	104–120 dB (A1 op amp)
Input current range	150 pA–160 µA (DC simulation range)	∼5 µA–750 µA (TIA full-scale, programmable)
Dynamic range	>5 decades (simulation supported)	∼2–3 decades (∼90 dB typ.)
Low-current accuracy	150 pA demonstrated in simulation; silicon validated within measure range	Performance dependent on gain configuration and noise floor

The urine experiments serve as proof-of-concept validation of the electrochemical readout architecture in a biologically relevant matrix. The study is not designed as a statistically powered clinical evaluation.

In addition, the wide current range of 150 pA to 160 µA is supported by DC simulation of the proposed architecture. Silicon measurements presented in this study validate functional operation and linear response within the experimentally tested concentration range under enzymatic sensing conditions. Exhaustive multi-decade noise characterization and minimum resolvable current analysis were not the primary focus of this study and remain areas for future investigation.

Accordingly, the comparison should be interpreted as an architectural analysis of electrochemical readout design strategies rather than a direct experimental performance benchmark between the proposed circuit and commercial AFE devices.

### 3.6 Limitations of the study

#### 3.6.1 Limitation of electrochemical validation

Although urine is a chemically complex matrix containing multiple electroactive species, this study did not include a systematic interference analysis using spiked compounds such as ascorbic acid, pharmaceuticals, or representative endogenous redox-active metabolites. In addition, while the applied oxidation potential (∼0.7 V) follows the established H_2_O_2_ detection principle and manufacturer-calibrated operating condition of the commercial strip, detailed voltammetric characterization to identify diffusion-limited plateau regions was not the primary focus of this study. Rather, its objective is to validate a CMOS potentiostatic readout architecture under enzymatic amperometric sensing conditions and not chemical optimization of UA assay selectivity. Comprehensive electrochemical interference and selectivity studies will be considered in future work to further strengthen analytical specificity.

#### 3.6.2 Dynamic range characterization limitation

While circuit-level DC simulations demonstrate that the proposed architecture can maintain stable RE bias and current replication across an input current span of 150 pA to 160 µA, the experimental measurements presented here focus on the UA sensing range associated with the enzymatic test strip used for urine validation. Consequently, a comprehensive experimental characterization of the full multi-decade current range—including minimum detectable current, noise floor analysis, and systematic load or impedance variation tests—was beyond the scope of the present study. These measurements would require dedicated low-current instrumentation and calibrated current injection experiments and will be addressed in future research to further quantify the ultimate detection limits and robustness of the architecture.

Nevertheless, the silicon measurements confirm stable potentiostatic bias regulation and accurate current readout within the experimentally evaluated UA concentration range (20–500 ppm), demonstrating the practical applicability of the proposed architecture for electrochemical biosensing.

### 3.7 Portable multi-parameter detection device

**FIGURE 11:**
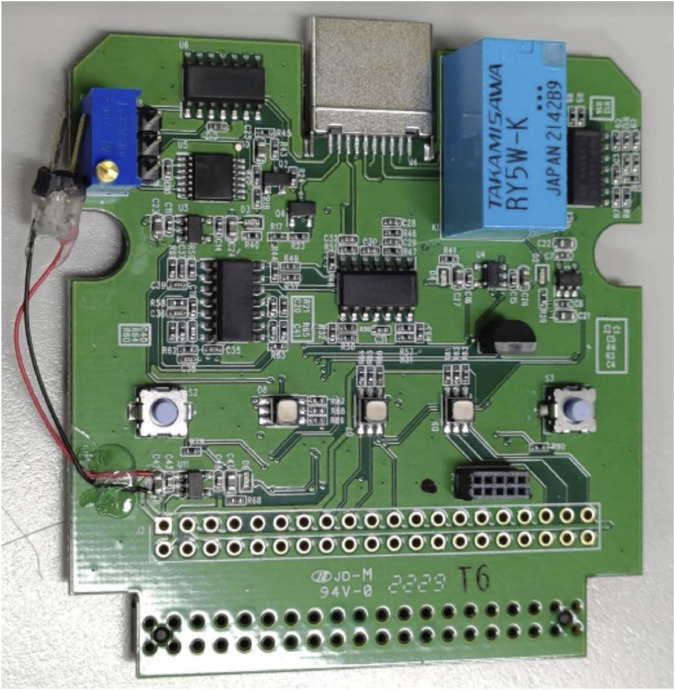
Portable multi-parameter detection PCB module.

**FIGURE 12:**
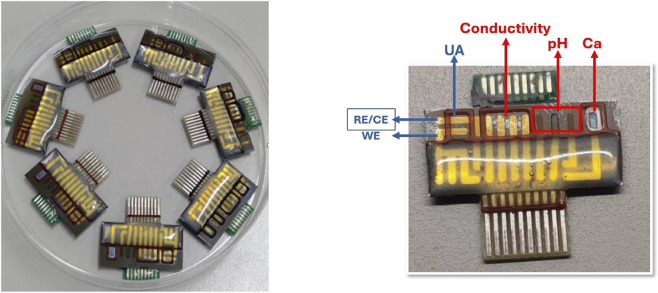
Integrated multi-parameter urine sensor (provided by Chang Gung University) incorporating an electrochemical cell for uric acid sensing together with pH, calcium, and conductivity sensing elements.

A portable multi-parameter detection platform for urolithiasis applications has been developed here. Although the UA readout IC based on Figure 1B architecture has been designed and characterized at the chip level, the current system platform implements the UA readout function using discrete components on a printed circuit board PCB, together with discrete front-end circuits for conductivity and Ca+/pH sensing (Figure 11). This board-level realization allows flexible system-level validation of the sensing architecture and signal processing chain prior to full IC integration. In parallel, the development of a unified sensor module capable of simultaneously sensing multiple urine parameters is ongoing, and the initial single-sensor prototype under evaluation (Figure 12) shows an integrated multi-parameter urine sensor provided by Chang Gung University, which incorporates a three-electrode electrochemical cell for UA amperometric sensing along with additional elements for pH, calcium, and conductivity measurement. During operation, the sensing channels are measured concurrently, and their analog outputs are digitized using an on-board microcontroller, followed by digital signal processing, data aggregation, and transmission to a cloud server.

## 4 Conclusion

This study presented the design, implementation, and experimental validation of a wide-dynamic-range amperometric readout circuit and sensing platform for urinary uric acid (UA) monitoring, targeting applications in urolithiasis risk assessment. A CMOS potentiostatic front end was developed to support both two- and three-electrode electrochemical sensors within a unified architecture, enabling flexible sensor interfacing without modifying the core readout circuitry. Implemented in a 0.18-µm CMOS process, the circuit supports a simulated sensing current range of 150 pA to 160 µA, corresponding to more than 5 decades of dynamic range, with relative error below 2% under DC analysis; silicon measurements confirm stable potentiostatic operation at the sensor interface. The active readout core occupies 102 µm × 195 µm and operates from a 3.3 V supply, demonstrating a compact solution suitable for portable electrochemical sensing systems.

System-level validation using urine samples demonstrated a linear response over a UA concentration range of 20–500 ppm, with measurement results showing functional agreement with the commercial analyzer within the tested range. Measurements from both PCB-based implementations and the fabricated silicon prototype confirm that the proposed architecture maintains linearity and stability across a wide range of electrochemical conditions. The separation of potentiostatic control and current readout through dual OTA feedback loops improves robustness against sensor impedance variations and transient effects, which is critical for reliable on-chip electrochemical interfacing and provides an alternative architectural approach to electrochemical readout compared with general-purpose commercial AFEs.

In addition to single-analyte sensing, the readout circuit was integrated into a portable multi-parameter urine sensing platform enabling concurrent measurement of UA, pH, calcium-related, and conductivity signals. This demonstrates the scalability and reusability of the proposed architecture for multi-channel biochemical sensing. Future research will focus on tighter system integration with embedded control and data processing to enable longer-term monitoring and data-driven analysis. With its compact silicon footprint, stable potentiostatic control, and an architecture capable of supporting multi-decade current sensing in simulation, the proposed readout IC provides a practical and extensible solution for integrated amperometric biosensing. Experimental results demonstrate reliable operation within the experimentally evaluated UA concentration range (20–500 ppm), while future research will focus on comprehensive experimental characterization of the architecture’s multi-decade current capability. The urine-based UA measurements presented here thus serve primarily as proof-of-concept validation of the electrochemical readout architecture in a biologically relevant sensing scenario rather than as a comprehensive electroanalytical characterization of assay selectivity in complex biological matrices. The experimental results presented here therefore validate the electrochemical interfacing capability of the proposed readout circuit within the tested sensing range, while the broader dynamic-range capability represents a simulation-supported architectural potential.

## Data Availability

The original contributions presented in the study are included in the article/supplementary material; further inquiries can be directed to the corresponding author.
